# Engineered phages for selective adsorption of rare earth elements

**DOI:** 10.1038/s41598-025-07604-3

**Published:** 2025-07-02

**Authors:** Alexander M. Ditzel, Scott N. Dean, Ellen R. Goldman, Jinny L. Liu

**Affiliations:** 1 Postdoctoral Fellowship for American Society for Engineering Education, Washington, DC, USA; 2 Center for Biomolecular Science and Engineering, US Naval Research Laboratory, 4555 Overlook Ave., Washington, DC, 20375 USA

**Keywords:** Engineered phages, Rare earth elements, Phage display, Tb, Peptide, Adsorption, FRET, Biological techniques, Materials science

## Abstract

Biomaterial extraction of rare earth elements (REEs) offers an environmentally friendly and efficient alternative to chemical solvent methods. M13 Phages showing less non-specific REE binding without glycosylated polysaccharides have multiple copies of p8 fusions to display peptides with selective binding for the desired REEs. In this study, we developed two diverse phage display libraries: one displaying 12-mer peptides and another displaying 17-mer circular peptides, each with over 1 × 10^7^ unique peptides. Six fluorescent protein-peptide fusions, CFP-peptide-YFP, were prepared for measuring K_d_. Their REE affinity, ranging from 2 to 30 micromolar (µM), were generally weak, but a novel peptide-phage, H11G-p8, a derivative of H11-p8, showed a better affinity toward Eu than others. Recombinant phages displaying H11G and E1 were also assessed for their K_i_ (phage inhibition) values, ranging 10^−11^ to 10^−12^ molar (M) resulting from the combination of specific binding to the REE and non-specific binding from the phage backbone. Both phages were further evaluated for direct REE binding as free phages in solution and as immobilized phages bound to resin. Both phages showed preferential binding toward Tb, with H11G-p8 consistently exhibiting higher specific Tb binding, indicating its greater potential for separating Tb from other REEs.

**Supplementary Information:**

The online version contains supplementary material available at 10.1038/s41598-025-07604-3.

## Introduction

Biomaterial extraction of rare earth elements (REEs) offers an environmentally friendly alternative to traditional mining and solvent extraction techniques, which can be harmful to the environment. Some of these biomaterials can be recycled and reused, making the process more sustainable^1,2^. Thus, this approach provides an efficient REE recovery method compared to traditional techniques, which are often energy-intensive and generate significant waste.

REE ions bind non-specifically to most negatively charged groups (such as –COOH, -OH, -SH) found on protein, phospholipids and polysaccharides present in most microorganisms, such as bacteria, and yeasts^3,4^. Various approaches achieve high specificity in separating REEs from non-specific bio-adsorption, with the most common being the use of specific REE-binding proteins, such as LanM (Lanmodulin), or peptides targeting the desired REE. Previous studies found that LanM expressed in methylotrophic bacteria, exhibits strong binding affinity to REEs with picomolar dissociation constants, without significant binding to other metal ions^5–7^. Each LanM contains four metal coordination motifs (EF hands), each capable of binding three metal ions^8^. Individual EF hands of 12 amino acid residues exhibit nanomolar (nM) to micromolar (µM) affinity, which are weaker than those of LanM^9^; however, peptides are very stable in extreme pH conditions, enduring multiple cycles of binding and acid elution when displayed on matrices.

Although LanM and EF hand peptides bind selectively to REEs, they lack selectivity for any specific REE. Thus, engineered protein/peptides or bioplatforms are needed. M13 phage display offers a powerful tool to select novel REE-binding peptides toward the desired REE. As M13 phages lack glycosylated polysaccharides, they offer a superior bioplatform with reduced non-specific binding. Additionally M13 phages provide the ability to display hundreds to thousands of peptide copies on each phage capsid through fusion to the major capsid protein, p8 (approximately 3300 copies per phage), resulting in peptide-phage with increased specificity and avidity to capture REEs more efficiently^10,11^. In fact, M13 phage display library displaying random peptides through tail protein, p3, was constructed in earlier studies to select Nd binders by quantifying phage amounts binding to immobilized Nd^12,13^. However, there was no data shown on the binding specificity toward other REEs in their studies. Several Tb binders were also identified by phage display screening against solid Tb (Terbium) mixture powder and resulting peptides were later used for separating Tb + 3 from other metals^14,15^.

Our previous research showed that genetically engineered bacteriophages displaying EF hands were able to selectively recover Nd from dilute aqueous solutions^11^. In this study, we developed two phage display libraries displaying diverse peptides, one featuring 12-mer peptides and the other featuring 17-mer peptides to screen potential LanM mimetic peptides against a panel of 4 REEs. We were able to obtain a list of potential REE peptides using phage competition assay. Down selected peptides were further evaluated using FRET assays and incorporated into phage genomes to create recombinant phages for K_i_ measurements. The most promising candidates were selected and further evaluated using REE direct binding assays with phages in solution and immobilized states.

## Results

### Diversity of peptide display phage library

Two main phage libraries were constructed. The 12-mer phage display library was created by randomizing 12 amino acids within an EF hand, and fusing them to p8. The 17-mer phage display library was derived from the circular TB2 peptide (ACVDWNNDGWYEGDECA) by fixing two cysteine residues and randomizing the non-cysteine residues (XCX_13_CX). Sanger sequencing of twenty clones from each library revealed that 50–60% contained full-length sequences without stop codons. Both libraries contain more than 1.0 × 10^7^ unique clones. Additionally, 250 bp amplicons from the 12-mer display library were sent out for high-throughput sequencing to confirm the diversity. Based on the analysis of 1 M reads, there were approximately 2.7 × 10^7^ unique clones, with 87% full-length clones calculated from the first 1000 most repeated sequences. The 1000 most frequent DNA sequences were obtained using the un-nest tokens function from the Tidytext R package, where ngrams of length 36 nucleotides were collected and sorted by frequency of appearance in the sequencing data. These top 1000 most frequent sequences were then translated to amino acid sequences in reading frame with p8 and the first top 20 sequences were shown in Table S2. To consolidate similar sequences, we employed fuzzy joining and alignment techniques, grouping and aligning closely related peptide sequences to generate consensus sequences for the peptides. The hgh-throughput sequencing results demonstrated the broad diversity of the phage library, which is ideal for selecting phages toward the desired targets, which were then used for biopanning and further characterization.

### Biopanning and selection of potential peptide-phages binding to Nd, Eu, Dy, and Yb

**Fig. 1:**
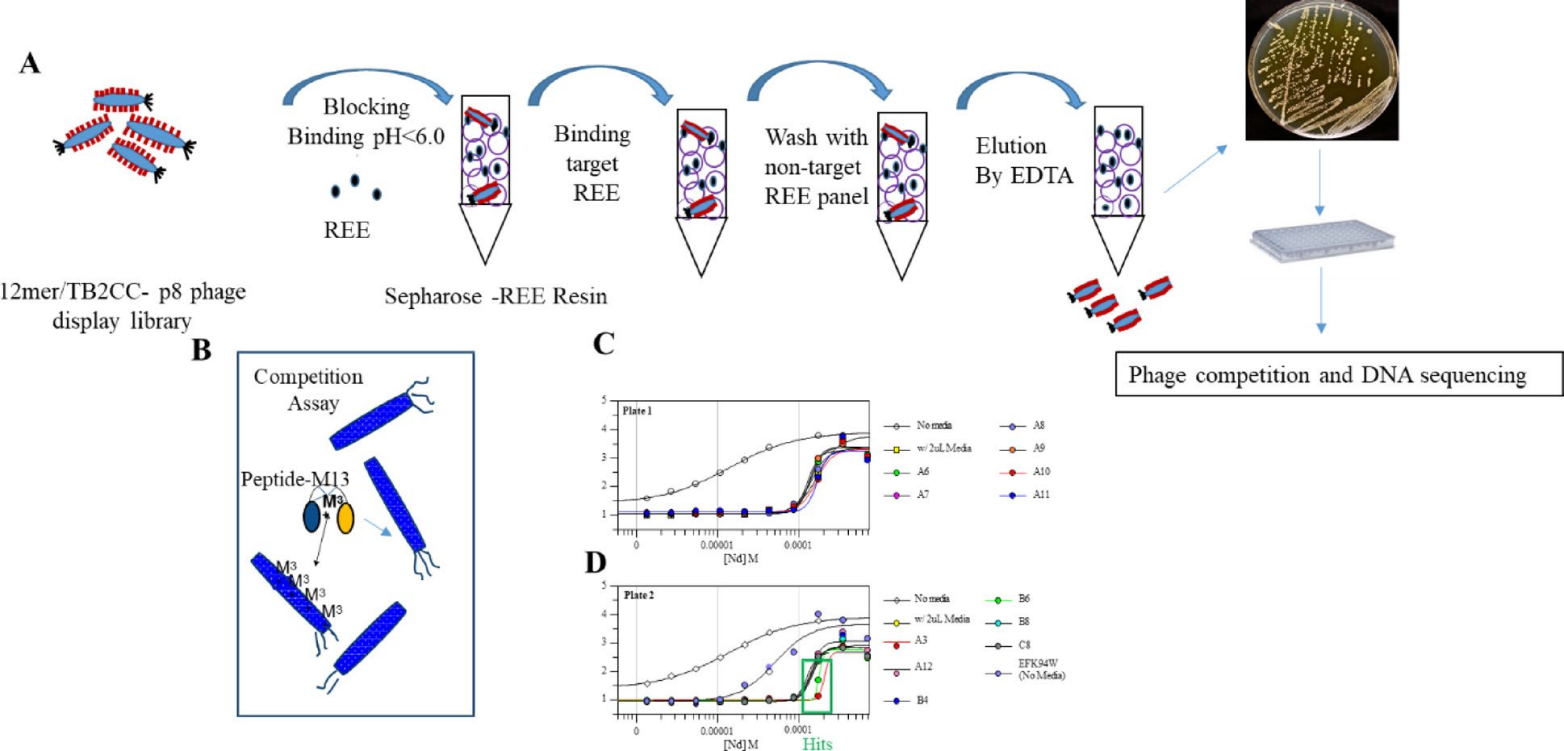
Schemes for phage bioanning and phage competition assay with Nd binding phages candidates from 96 well plate. (**A**) Phage library biopanning to obtain 1^st^ batch of potential binders. (**B**) Mechanism of FRET competition assay to further screen the peptide-phage binders. (**C**) Peptide display phages exhibited similar IC50 curves. (**D**) A different IC50 curve exhibited in the presence of EF3K94W phage, resulting from the competition of REE binding with EF3 fluorescent protein fusion and A3-p8 and B6-p8 also showed different binding curves indicating they might be potential binders. Potential binders were reassessed using a large amount of the phages.

The R1 elution for immobilized Nd resulted in 6.0 × 10^8^ pfu after EDTA elution (Fig. 1A). Ninety-six to 192 colonies were picked from the plates to prepare monoclonal phages for the phage competition assay (Fig. 1B). The REE binding curve for each monoclonal phage was compared with the negative control (media) and a positive control, (EFk94W (DPDNDGTLDKWE) phage) in Fig. 1C,D. Two rounds of binding evaluation were performed to select potential binders against Nd, Eu, Dy, and Yb. The nomenclature for the potential binders were labeled with the screened REE first followed by the position in 96 well plate, such as NdH1, etc. We selected 15 unique sequences against Nd and Eu from the 12-mer library (Table S3) and 17 unique circular peptides against Dy, Nd, and Yb from the 17-mer library (Table S4) based on the phage binding curves with media and control phages. At least two individual tests were performed. For the second test, a new phage preparation from 30 mL cultures was used to reassess their binding curves for down-selection.

### Measurements of Kd for peptides using peptide-fluorescent protein fusions

**Fig. 2:**
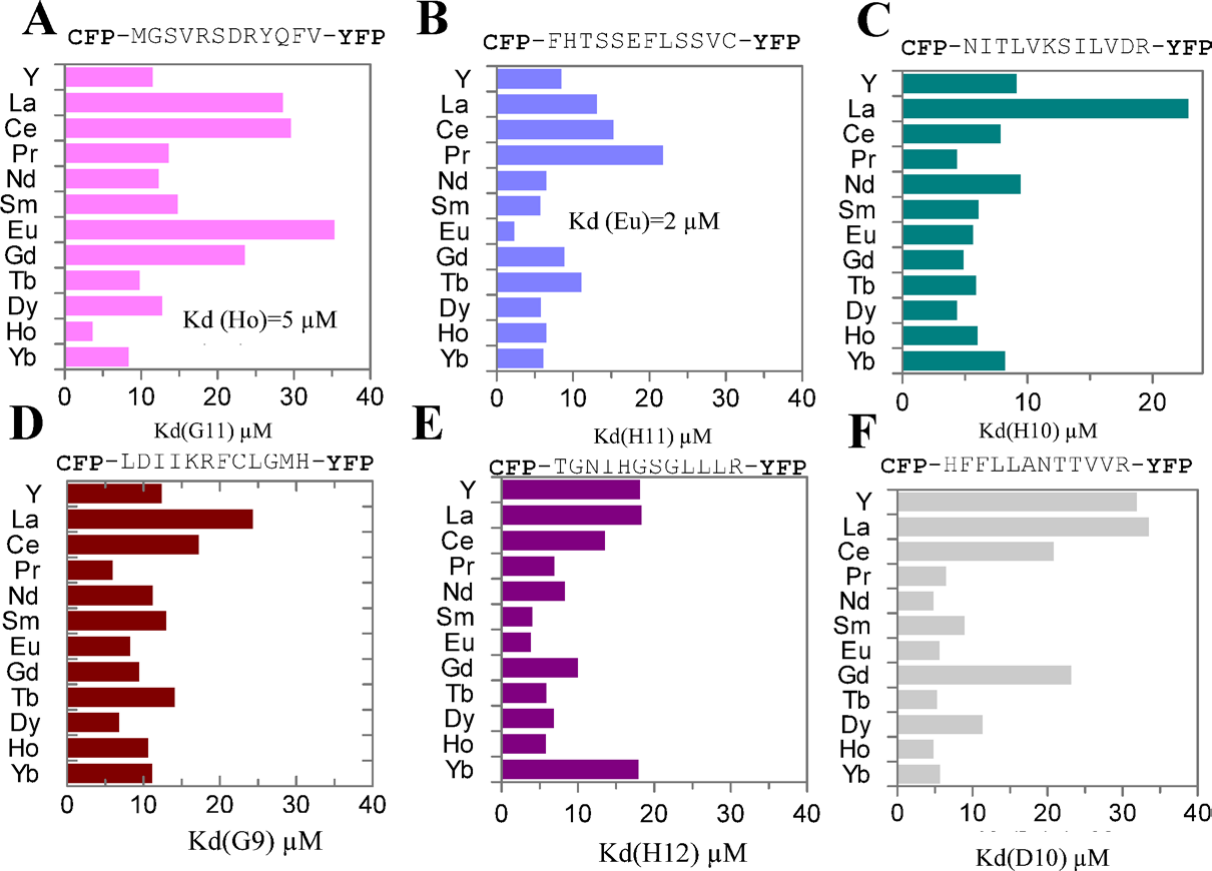
K_d_ measurements for a panel of 12 REEs using peptide-fluorescent fusions. Six peptides selected against Nd from 12 mer phage display library were inserted between CFP and YFP (**A**–**F**) to measure K_d_ for each REE. The K_d_ values for each REE were plotted and compared in A-F panels.

Six peptides screened against Nd (Table S3) showing the best binding curves were clone to create CFP-peptide-YFP constructs as described in the methods section. The fusions were then mixed with each REE at various concentrations to measure the ratio of emission of 527 to emission of 470 to measure K_d_, which were plotted together and compared as indicated in Fig. 2. All six peptide-fluorescent protein fusions showed binding to all 12 REEs with K_d_ values ranging from 2 to 30 µM. We observed that H11 (aka NdH11) showed tenfold higher affinity toward Eu than Pr (Fig. 2B). In contrast, the G11 peptide showed higher affinity for Ho compared to other REEs (Fig. 2A). The other four peptides do not exhibit specific binding toward certain REEs (Fig. 2D,F). These selected peptides were then used to create recombinant phages with enhanced REE-binding properties.

### Preparation of recombinant phages and Ki measurements

**Fig. 3:**
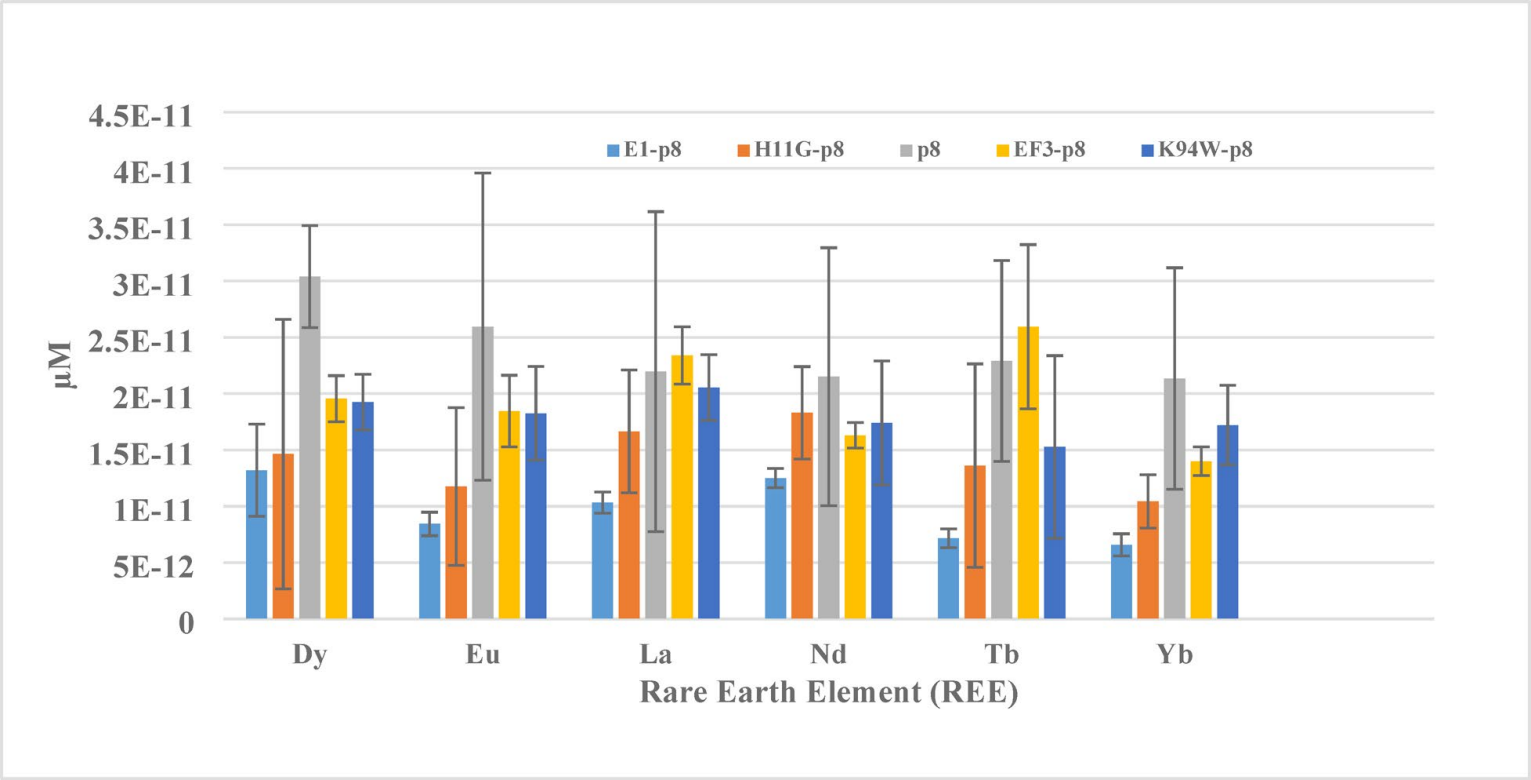
Phage Ki measurements for 6 REE panel. Novel recombinant phages, E1-p8 and H11G-p8 phages were compared to a positive control phage, EF3-p8 and negative control phage, p8. Biological and operation replicates were used for obtaining STDEV (SD).

The insertion of exogenous DNA into recombinant phage genomes were confirmed by Sanger sequencing. The integrity for purified recombinant phages were also assessed by passing through native agarose gel electrophoresis followed by staining with DNA dye, SYBR safe dye (Fig. S1A). The same gel was subsequently stained with Coomassie Blue (Fig. S1B). The preparation of the recombinant phages did not require helper phages, and the yield for bench-scale production could reach 2 g/L or higher, which is approximately 20-fold higher than that of display phages (unpublished results). To reduce batch variation, we purified phages using PEG precipitation combined with a tangential flow filter system to remove DNA, contaminated proteins, and cell debris, reducing the A260/A280 ratio to 1.09 (Fig. S1). Most of the recombinant phages were selected from the 12-mer library including E1 and H11G, a H11 variant replacing Cys with Gly, peptides expressed through p8 fusion as E1-p8 and H11G-p8 phages. These recombinant phages were used for measuring Ki for phages to compete for binding with six REEs: Tb, Yb, Nd, La, Dy, and Eu, using CFP-EF3-YFP as a template in the assay (Fig. S2 and Table S5). The down-selected recombinant phages with the lowest Ki among the six REEs were compared with p8 phage as a negative control and EF3 (DPDNDGTLDKKE)-p8 phages as a positive control (Fig. 3). Our results indicated that negative control phage, p8, exhibited the highest Ki values for all six REE among the four phages. EF3-p8 phage displaying EF3 peptides showed lowest Ki for Yb among six REEs. E1-p8 and H11G-p8 phages in general showed lower Ki than p8 and EF3-p8 phages, but E1-p8 phage showed the lowest Ki, especially Tb and Yb, for all 6 REEs among the four phages. The Ki measurement is based on the fitted model with 4 parameters as indicated in Fig. S2. Although we have cloned and prepared 10 of 17-mer recombinant phages listed from Table S4, we did not see significantly lower Ki than E1-p8 and H11G-p8 phages (Data not shown).

### Membrane filtration of unbound REE

**Fig. 4:**
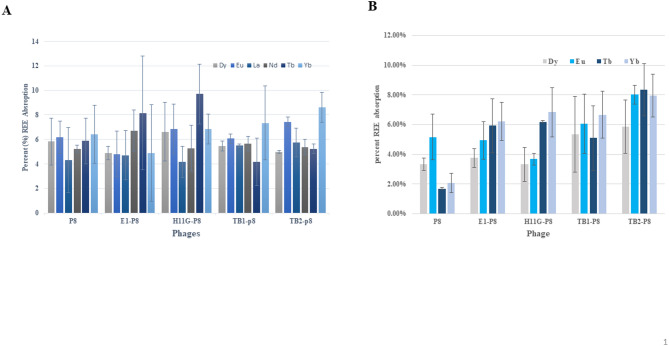
Phage direct binding assay using membrane filtration. (**A**) Binding assay was performed at pH5.5 with 4 recombinant phages expressing E1, H11G, TB1 and TB2. TB1-p8 and TB2-p8 phages are positive controls and p8 phage is negative control. (**B**) The same binding assay was also conducted at pH6.0.

REE direct binding to phages was conducted by both free phages in solution and immobilized phages. Free recombinant phages in solution were used for the first direct binding assay in 50 mM MES at pH 5.5 and pH 6.0, followed by filtration through a 50–100 kDa membrane filter (Fig. S3A). The unbound REEs passed through the filter, and the bound REEs were retained. The p8 control phage showed 4–6% non-specific binding for all six REEs, while H11G-p8 phages showed more specific binding toward Tb (approximately 10%) at pH 5.5 (Fig. 4A).At pH 6.0, error bars for binding were lower toward Tb and Yb in both E1-p8 and H11G-p8 binding in both Tb and Yb (Fig. 4B), suggesting both recombinant phages exhibited preferential binding toward heavy REEs (Tb and Yb). TB2-p8 phages also demonstrated increased binding toward heavy REEs, Tb, Yb, and Eu. Data for Nd and La could not be obtained at pH 6.0 due to precipitation (Fig. 4B).

### REE binding to immobilized phages

In the second direct binding assay, immobilized phages were used (Fig. S3B). A total of 4 mg of phages were conjugated to 0.25 mL of NHS-activated Sepharose resin. The REE solutions were passed through a column packed with the phage-conjugated Sepharose. Our results indicated that Tb binds to immobilized H11G-p8 at pH 5.5, showing 6% specific binding affinity toward Tb (Table1).

**Table 1:** Tb binding to immobilized phages on Sepharose.

Conjugated phage	Total binding (%)	Phage binding	STDEV
Starting	0.00		
Blank	24.44	0.00%	0.30%
P8	25.57	1.13%	0.95%
H11G-p8	30.17	5.73%	1.20%
E1-p8	29.03	4.59%	1.26%

### Structural analysis of p8 fusion variants

**Fig. 5:**
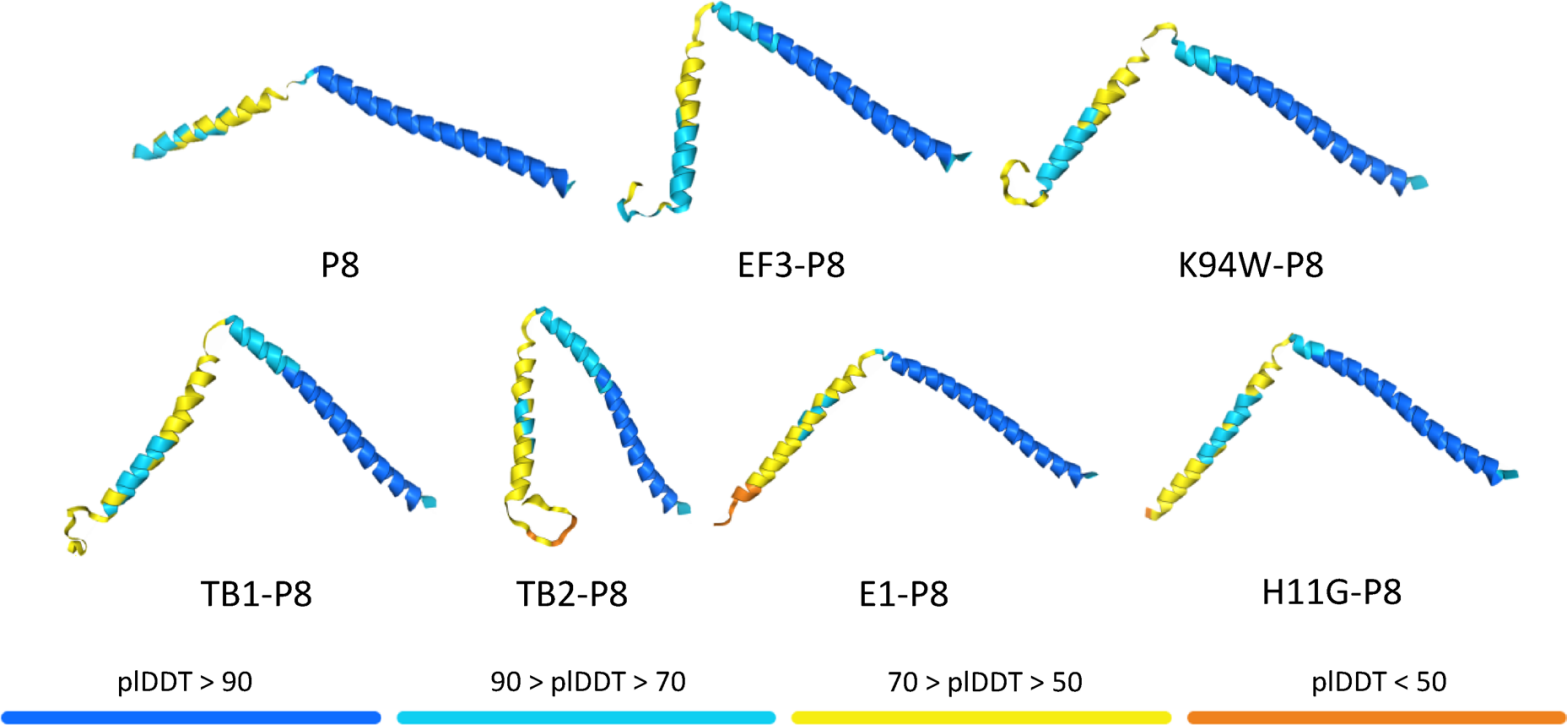
Structure models for peptide-p8 fusion monomer by alpha fold 3. The color shows the confidence level of modeled regions.

Alphafold 3 was used to predict the structure as indicated in Fig. 5.

## Discussion

We observed substantial non-specific binding in non-glycosylated phages during bio-panning, leading us to implement several strategies to enhance specificity and reduce non-specific REE binding. (1) Reduced background binding by washing bound phages with other non-targeted REEs in the presence of 1% Tween. We did observe that some target binders had highest curve shift in phage competition assay. (2) Increased high affinity binders by the construction of a circular peptide phage display library. Previous studies showed that the Tb (Terbium) binding peptide 2 (TB2: ACVDWNNDGWYEGDECA) exhibits nanomolar affinity toward Tb, 100-fold higher than for other REEs. This higher binding affinity may result from the formation of a disulfide bond between two cysteines (Cys), positioning Tb closer to the peptide. We therefore randomized the residues other than those two Cys to develop higher affinity binders toward other REEs; however, we did not see any improvement for the binding selectivity in peptide recombinant phages compared to the 12 mer randomized peptide display library. (3) Validated the binding curve shift by biological replicate and triplicate. Phage-displayed candidate peptides were prepared independently at least twice and evaluated for their REE binding curves multiple times by comparing with the control phages. The meticulous preparation of phages was aimed at reducing batch variations to obtain consistent phage competition results.

By analyzing shifts in phage binding curves, we identified the top six phage-displayed peptides towards Nd binding. These peptides were subsequently fused to CFP and YFP. Fluorescence resonance energy transfer (FRET) from YFP to CFP upon fusion peptide binding to REE revealed that all six peptides exhibited micromolar (µM) affinity toward most REEs, comparable to previously published peptides^16^. Although these peptides did bind to Nd, we did not see good selectivity (tenfold less K_d_ values than other REEs). We noticed that H11 demonstrated the lowest K_d_ toward Eu and G11 toward Ho among a panel of 12 REEs. The discrepancy in preferential REE binding between peptides and peptide-displayed phages is likely due to the charges of backbones and the configuration of assembled peptides displayed on phages.

Our peptide’s affinity for REEs in the micromolar (µM) range was relatively weak compared to the picomolar (pM) affinity of LanM^6,17^. To enhance binding affinity via increased avidity, others immobilized multiple copies of peptides onto a substrate or matrix, which improved selectivity after multiple binding and extraction cycles^18^. Using a similar strategy, we employed phage as a matrix or bio-platform to display multiple peptide copies, aiming to increase avidity. This approach also offers the advantage of peptide expression and assembly into phages without requiring purification, which is particularly beneficial for cysteine-circular peptides that often have low expression yields. From the 12-mer phage display library, we successfully identify two novel peptide phages, H11G-p8, and E1-p8, with lower Ki values compared to the other phages. Due to challenges in expressing and purifying circular peptides, we directly cloned sequences for recombinant phage creation rather than constructing circular peptide-fluorescent protein fusions for Ki measurements. Ki measurements revealed that circular peptide phages generally exhibited higher values than TB2-p8 phages, indicating none surpassed the parental TB2 peptide’s binding affinity. This finding suggests that specific residues between the two cysteines play a more critical role in binding than in forming a pocket to trap REEs. A comparison of all published Tb-binding peptides—TB1 (YIDTNNDGWYEGDELLA), TB2 (ACVDWNNDGWYEGDECA), Terbofluor (DKNADGWIEFEEL), and LBT (GYIDTNNDGWIEGDELY)—highlights a common Trp (W) residue in Tb coordination.

For the REE direct binding assay, we evaluated two novel recombinant phages, H11G-p8 and E1-p8, alongside control phages. Non-specific binding was observed in both assay formats. Free phage backbones exhibited 4–6% non-specific REE binding, while immobilized phages on sepharose resins displayed 25% non-specific binding (Table 1), likely due to the hydroxyl and carboxyl functional groups on the resin. After correcting for non-specific binding, E1-p8 and H11G-p8 demonstrated approximately 4% and 6% specific Tb binding, respectively, indicating a preference for Tb over other REEs. The specificity toward Tb is likely conferred by the displayed peptides. Specifically, H11G (FHTSSEFLSSVG), which contains both glutamic acid (Glu) and histidine (His), likely facilitates strong coordination with Tb, along with weaker hydroxyl group coordination via serine (Ser) residues. In contrast, E1 (MGQPRVLVRHVV) contains only histidine residues for Tb coordination, resulting in a lower affinity than H11G. Interestingly, TB1-p8 and TB2-p8 phages showed greater affinity for Yb than Tb at pH 5.5 compared to pH 6.0, highlighting the significant impact of pH on Yb and Tb binding to phages. Overall, selective binding to Tb at pH 5.5 was consistently observed for H11G-p8 in both phage binding assays. In contrast, TB2-p8 and E1-p8 exhibited only modest Tb preference relative to the p8 control phages.

Based on selectivity and pH evaluation, a multi-cycle Tb enrichment process can be developed using column chromatography with beads (e.g., agarose, polystyrene) immobilized with H11G-p8/E1-p8 phages, as described by Medin et al.^19^. Once this enrichment process is optimized, the setup can be scaled up to obtain larger quantities of Tb. While phages without glycosylation offer reduced non-specific binding compared to bacteria and spores (due to their surface glycans, sugars, and polysaccharides), they may exhibit higher background binding than proteins and peptides. Indeed, peptide-conjugated beads packed into columns have also been demonstrated for multi-cycle enrichment of rare earth elements (REEs), suggesting alternative approaches^2^.

We summarize the following factors influencing REE binding to phages: (1) pH: Functional group ionization and REE solubility vary with pH. TB1-p8 and TB2-p8 phages exhibited different affinities for Yb at pH 5.5 and 6.0. Nd and La binding were not observed due to REE insolubility under experimental conditions. (2) Ionic strength: Competing ions affect REE binding by occupying binding sites. Washing with Tween 20 mitigated non-specific binding. (3) Phage surface properties: Surface variations among recombinant phages significantly affected REE binding, as seen with H11G-p8 and E1-p8. (4) REE ionic radii and charge density: Smaller ionic radii and higher charge densities contributed to stronger binding. For example, Tb showed preferential binding to H11G-p8 and E1-p8 at pH 5.5 due to these properties.

Alpha Fold 3 structural predictions of our p8 fusion variants revealed interesting conformational differences that may explain their varying binding properties. While these predictions are limited by representing only monomeric forms without the context of the complete viral capsid assembly, they provide valuable insights into potential structural arrangements. Most fusion peptides (EF3-p8, EF3K94W-p8, E1-p8, TB1-p8, and TB2-p8) were predicted to form flexible loop regions at the N-terminus of p8, consistent with typical surface-exposed peptide additions. Notably, H11G-p8 displayed a distinct structural feature where the fusion peptide appeared to continue the existing N-terminal α-helix rather than forming a loop region. This extended helical conformation may contribute to H11G-p8’s enhanced binding specificity and affinity for Tb, possibly by providing a more rigid and well-defined coordination environment for the REE. However, future experimental structure determination would be necessary to validate these predictions and understand how the viral capsid context might influence the final conformations of these fusion proteins.

## Conclusion

The M13 phage display system is a powerful tool for selecting REE-specific peptides and serves as an excellent bioplatform for displaying multiple peptide copies to enhance REE binding. In this study, we identified two novel phages, H11G-p8 and E1-p8, which express multiple copies of selected peptides and demonstrated preferential binding to Tb in direct binding assays, both in free phage and immobilized configurations. Among these, H11G-p8 exhibited superior specificity compared to E1-p8, TB2, and p8 control phages. This enhanced specificity positions H11G-p8 as a promising candidate for REE enrichment or extraction from diverse feedstock sources.

## Materials and methods

### Reagents and chemicals

All enzymes, M13KO7 helper phages, and M13Mp18 RF vector required for recombinant phage cloning, PCR and plasmid cloning were purchased from New England Biolabs (Ipswich, MA). All chemicals, antibiotics and Terrific broth-Novagen described in the protocol were acquired from MilliporeSigma (St. Louis, MO). Bacterial cell culture media, LB and SOC, were purchased from Fisher Scientific (Waltham, MA). The DNA gel extraction kit and PCR purification kit were purchased from Qiagen (Germantown, MD).

### Construction of peptide display M13 phage libraries (12 mers, 17 mers with circular peptide, TB2CC-pVIII)

To construct the libraries, we used pecan 21 phagemid vectors, a gift from Dr. Andrew Hayhurst (Texas BioMed Lab, San Antonio, TX), inserting P8-peptide DNA fragments according to our established protocol^20,21^. In brief, DNA encoding peptide-p8 were amplified using forward primers (Random 12mers F, and TB2CC F) and the reverse primer p8 Rev, as listed in Table S1. The amplified DNA fragments, which are 292 bp in size, were first purified using the Qiaquick PCR kit. These purified fragments and pecan 21 were then digested with NcoI and EcoRV for 2 h at 37 °C. The digested fragments were separated and purified from a 1% Tris acetate agarose gel using the Qiaquick gel purification kit and Qiaquick PCR kit. The resulting p8 fusion DNA fragments and pecan 21 backbone fragments were ligated at 16 °C for 18 h using T4 DNA ligase, each 100 ng of ligated products was then electroporated into 40 µL of XL1-Blue electrocompetent cells (Agilent Technologies, Savage, MD) and resuspended in 1 mL of SOC medium immediately after electroporation. Approximately 5 µg of the ligated product was electroporated and resuspended into 50 mL SOC. Every 15 mL of SOC medium was spun down and resuspended in 3 mL of SOC, then plated onto a Bioassay Dish plate (245 mm × 245 mm) and incubated at 37 °C overnight. The next day, the bacterial lawn was scraped into LB supplemented with 2% glucose, 100 µg/mL ampicillin, and 15% glycerol. The resulting bacterial resuspension was then frozen in dry ice and stored at -80 °C.

### Measurements of phage library titters

To calculate the titer of the library, 1 µL was taken from 3 mL of the bacterial suspension before plating and tenfold serial dilutions were made. A 100 µL dilution was plated from the -5 (10^fivefold dilution), -6, -7, and -8 tubes. To measure the diversity of sequences, twenty or more colonies were randomly picked and grown to isolate phagemid DNA and sequenced using Pecan 21 seq F (Table S1).

### Preparation of peptide display phages from M13 phage display libraries

To prepare display phages, 100 µL of scraped bacteria were grown in 50 mL LB supplemented with 2% glucose and 100 µg/mL ampicillin at 37 °C for approximately 2 h until the absorbance at 600 nm was around 0.6 to 0.8. Ten mL of these log-phase bacteria were mixed with the proper amount of M13K07 to reach a multiplicity of infection (MOI) of 20. The mixture was incubated at 37 °C for 30 min before spinning at 6000 × g for 10 min to pellet the infected cells. The infected cells were then resuspended into 50 mL of LB supplemented with 50 µg/mL kanamycin and 100 µg/mL ampicillin and grown shaking at 225 rpm in a 30 °C incubator for 24 h. The next day, the supernatant was collected after spinning at 15,000 × g for 15 min. One-fifth volume of 20% Polyethylene Glycol (PEG) 8000 and 2.5 M NaCl (final concentration 4% PEG and 0.5 M NaCl) was added to the supernatant to precipitate the phages on ice for at least one hour before centrifuging at 15,000 × g for 15 min. The phage pellet was resuspended in 5 mL of 50 mM HEPES pH 7.4 and centrifuged at 15,000 × g for 10 min to remove cell debris. The supernatant was measured at A280 to estimate the concentration of phage for further application. Alternatively, phage concentrations were measured by the BCA method. Phage titers were estimated based on a factor of 3.69 × 10^13 per mg of protein.

### Analysis of display peptide sequences by high-throughput sequencing

100 µL of M13 phage display bacterial stock was grown in 50 mL LB supplemented with ampicillin at 37 °C overnight. The next day, plasmid DNA was isolated from the bacterial culture using a Midi prep kit (Qiagen, Germantown, MD). The resulting plasmid DNA was used as a template for PCR to obtain amplicons (250 bp) containing DNA fragments encoding random peptides and partial p8. Two primers, NGSsequF and NGSsequR1 (Table S1), were used to generate the amplicons. The amplicons were separated and purified from 1% TAE gel using Qiagen kits as described above and sent out to GeneWiz (Azenta Life Sciences) for high-throughput sequencing.

### Biopanning with REE adsorbed on sepharose resins

Sepharose (MilliporeSigma) resins were treated with 0.1 M EDTA first to chelate the metals and washed with water. The chelated sepharose resins were resuspended in water for use. To prepare Nd/Yb/Dy/Eu-sepharose resins, 0.4 mL sepharose resins were packed into a small column, washed with 5 mL 50 mM MES pH 5.5, and then mixed with 0.5 mL 100 µg/mL NdCl_3_/DyCl_3_/EuCl_3_ (168 µM) and Yb(NO3)_3_ at room temperature for 30 min. The resins were then washed with 5 mL of 50 mM MES pH 5.5 before being blocked with 2–4% BSA (Bovine Serum Albumin) in 50 mM MES pH 5.5 for 30 min. After blocking, 0.5 mL M13 phages (0.5 mg/mL with approximately 2.5 × 10^12^ plaque-forming units) with 2–4% BSA, 2% Tween 20 in 50 mM MES pH 5.5 was added to the sepharose and shaken for 1 h at 25 °C. The sepharose resins were washed with 10 mL of 2% Tween 20 in 50 mM MES pH 5.5, followed by 10 mL of 50 mM MES pH 5.5 and 3 mL of non-target REE (100 mM for each element) before eluting with 2 mL 0.1 M EDTA. The elution was then concentrated and washed with 50 mM HEPES pH 7.4 using a 50 kDa Amicon Ultra-0.5 mL device (MilliporeSigma). Eluted phages were titrated and plated onto LB plates supplemented with glucose and ampicillin. All of the eluted phages were plated onto a Bioassay dish to make the R1 (Round 1) library.

### Monoclonal phage production

To prepare monoclonal phage, individual colonies from the eluted phage plates were inoculated into 150 µL LB containing 100 µg/mL ampicillin and 2% glucose in 96-well plates and grow with shaking (250 rpm) overnight at 37 °C. The next day, 25 µL of bacterial culture was transferred into a second 96-well plate containing 200 µL of LB with 100 µg/mL ampicillin and 2% glucose per well. The plate was grown shaking at 37 °C for 2 h. Glycerol stocks of the original 96-well plate were made by adding glycerol to a final concentration of 15% and stored at − 80 °C. To each well in the second plate, 25 µL LB containing 100 µg/mL ampicillin, 2% glucose, and 10^9^ pfu M13KO7 helper phage were added and allowed to stand for 30 min at 37 °C, followed by shaking for 1 h at 37 °C. Cells were spun at 1800 × g for 10 min, and the supernatant was aspirated off. Subsequently, 200 µL LB containing 100 µg/mL ampicillin and 50 µg/mL kanamycin was added to each well, and the plate was grown shaking overnight at 30 °C. The next day, the plate was spun at 1800 × g for 10 min, and the supernatant was used for the REE binding assay. Sixty-four colonies were grown to measure the K_d_ curve for fluorescent protein binding to Nd ions.

### Measurement of metal dissociation constants for fluorescent protein

The ratiometric method was used to measure the dissociation constants (K_d_) of each peptide. Metal standards for ICP-MS were titrated across the plates. Metal ions (5 µL) were titrated into dilute solutions (45 µL) of CFP-EF3-YFP (0.2–0.4 µM) and the emission ratio (r) was measured using 96-well black half-area plates.

1 $$r= \frac{(ex=434,em=527)}{(ex=434, em=470)}$$

End-point reads were done after 15 min and after overnight incubation. Emission ratio changes were plotted against total concentration of the titrant.

2 $$\frac{R-{R}_{min}}{{R}_{max}-{R}_{min}}=\frac{[{M}_{f}]}{{K}_{d}+[{M}_{f}]}$$

where R_min_ and R_max_ correspond to the emission ratios of the FRET substrate in the absence of ligand and at saturation. [M_f_] corresponds to the concentration of free metal. Data were fit to Eq. (2) using Grafit 5 (Erithicus Software Ltd.).

### Peptide screening using phage competition assay and K_i_ measurements

Prior to assays, phages were dialyzed against 50 mM MES buffer (pH 6.0) to measure the Ki binding constant between phage and REE ions in the presence of monoclonal phage supernatant. The FRET substrate CFP-EF3-YFP (0.2 µM) was mixed with varying concentrations of phage. These CFP-EF3-YFP/phage mixtures underwent serial 1:1 dilutions with water across the plate, followed by the addition of metal solution (5 µL, approximately 60 µg/mL) to each row, yielding a final volume of 50 µL per well. The assay plate included negative controls (no phage and media only) and a positive control (EF3-p8 phages) to determine the IC50 shift in the presence of CFP-EF3-YFP and metal, thereby enabling calculation of the Ki binding constant between phage and REE metal. All reactions were performed in 50 mM MES buffer (pH 6.0). Ki values were determined at each phage concentration by fitting to a 4-parameter logistic equation representing competitive binding using the curve fit function from Scipy using the known values of the phage concentration and metal concentration, and the bound/free ratio measured by the FRET signal.

### Construction and preparation of recombinant M13 phage

DNA encoding selected REE peptides with higher affinity and specificity were cloned into M13 phage genomes using the M13Mp18 RF vector according to the manufacturer’s protocol (New England Biolabs). In brief, M13Mp18 RF and peptide-P8 fusion DNA were cut with EcoRI and HindIII, ligated together, and transformed into competent JM01 cells. The mixture was shaken at 30 °C for 30 min. The supernatant was then titrated and plated on an XL1-Blue lawn. Three to five plaques were used as templates to amplify 500 bp fragments for Sanger sequencing (Eurofins Genomics, Louisville, KY) using primers M13 MCS F1, MCS Rev, and MCS seq F1 listed in Table S1.

### Preparation of recombinant M13 phage

A single colony of XL1-Blue was grown overnight in TBT (Terrific broth with 15 µg/mL tetracycline) at 37 °C, shaking at 250 rpm. The next day, 5 mL of the overnight culture was added to 50 mL TBT with anti-foam (1% polypropylene glycol) and shaken at 250 rpm for approximately 1.5–2 h to reach an A600 of 0.6–0.8. The phage at an MOI of 0.1–1 was added to infect the bacteria. The culture was incubated at 37 °C for 5 min and then switched to 30 °C, shaking at 250 rpm for 22 h. Cells were spun at 20,000 × g for 1 h, and the supernatants were filtered sequentially through 0.8 µm, 0.45 µm, and 0.22 µm filter membranes. The filtrate (250 mL) was then precipitated by PEG and NaCl at 4 °C overnight. The precipitate, after spinning at 15,000 × g for 15 min, was resuspended in 50 mM MES and purified through the Cogent Lab 150 tangential flow filter system (TFF) (MilliporeSigma) using a 300 kDa cutoff cartridge by washing with 1 L of 50 mM MES pH 6.0 buffer. The concentrated filtrate, approximately 15 mL, was then dialyzed in 1 L of 50 mM MES buffer and 1 g of Chelex 100 resins at 4 °C overnight. 0.05% sodium azide was also added to the buffer to prevent phage degradation. The protein concentration was measured at A280, with an A260/A280 ratio of 1.08–1.10.

### Direct binding assay-membrane filtration

The binding interaction between REEs and phage was assessed using a direct binding setup as indicated in Fig. S3A. Briefly, 300 µL of REE solution (95 µg/mL in pH 6.0, 50 mM MES buffer) was combined with 20 µL of phage solution (20–30 µg/mL in the same buffer) and incubated at 25 °C for 20 min to facilitate binding. The mixture was then passed through a 100 kDa molecular weight cutoff filter by centrifugation at 12,000 × g for 5 min or until complete passage of liquid. The flow-through containing unbound REE was collected for subsequent analysis. The retained material was washed twice with 300 µL of pH 6.0, 50 mM MES buffer, with each wash collected separately after centrifugation under the same conditions. Sequential elutions were performed using 300 µL of pH 4.3, 40 mM citrate buffer followed by 300 µL of pH 1.7 glycine buffer, with centrifugation steps as described above. Finally, two additional washes with 300 µL of 50 mM MES buffer at pH 6.0 were performed to restore neutral conditions.

### Xylenol orange assay for REE quantification

REE concentrations were determined using a xylenol orange (XO) colorimetric assay^22^. A working solution of 0.3 mM XO was prepared fresh. Standard curves were generated using REE concentrations of 0, 6, 12, 20, 30, and 40 µg/mL. Experimental samples from binding assay flow-throughs were diluted fivefold with pH 6.0 MES buffer prior to analysis, while wash fractions were analyzed without dilution. For the assay, 10 µL of sample was combined with 90 µL of XO working solution in microplate wells. The plate was subjected to 30 s of shaking, followed by a 10-min incubation period and an additional 30-s shake. Absorbance measurements were taken at 580 nm, and REE concentrations were calculated using the standard curve.

### Phage immobilization onto sepharose resins-deactivation and washing procedure

The conjugation protocol was performed by following manufacturer’s protocol for NHS Sepharose 4 Fast flow resins (Millipore Sigma) as indicated in Fig. S3B. In brief, NHS activated sepharose resins were mixed with 2 mg of recombinant phage at 25 °C overnight. Next day, excess active groups were deactivated using an alternating buffer wash protocol. The blocking buffer consisted of 500 mM ethanolamine and 500 mM NaCl at pH 8.3, while the wash buffer was 50 mM MES at pH 5.5. The protocol comprised sequential injections in the following order: (1) 3 × 2 column volumes (CV) of blocking buffer, (2) 3 × 2 CV of wash buffer, (3) 3 × 2 CV of blocking buffer, followed by a 30-min standing period, (4) 3 × 2 CV of wash buffer, (5) 3 × 2 CV of blocking buffer, (6) 3 × 2 CV of wash buffer, and finally (7) 2–5 CV of wash buffer to equilibrate the resin.

### REE-sepharose binding

The interaction between REE solutions and phage-immobilized resin was evaluated using a column chromatography approach at room temperature, with one CV defined as 250 µL. The complete buffer series was performed in the following sequence: (1) Column equilibration with 3 × 2 CV of pH 5.5 MES buffer, followed by aspiration with air to clear wash buffer; (2) Application of 1 × 2 CV of 100 µM REE binding solution in pH 5.5 MES, followed by aspiration with air to clear binding solution; (3) First wash series with 2 × 2 CV of pH 5.5 MES containing 1% Tween detergent; (4) Second wash series with 2 × 2 CV of pH 5.5 MES without Tween, followed by aspiration with air to clear wash buffer; (5) Elution with 2 × 2 CV of pH 1.7 glycine buffer, followed by aspiration with air to clear elution buffer; (6) Final wash with 3 × 2 CV of pH 5.5 MES; and (7) Storage in 4 CV of MES buffer. REE concentrations in flow through and wash fractions were quantified using the xylenol orange assay described above.

### Structural prediction of p8 fusion peptides

We employed AlphaFold 3 via the DeepMind server to predict the structures of various p8 fusion peptides. Structural predictions were generated for the parental p8 protein and six fusion constructs: H11G-p8, E1-p8, TB1-p8, TB2-p8, EF3-p8, and EF3K94W-p8. Each protein sequence was modeled as a monomer, representing a single unite of the viral capsid protein with its N-terminal fusion. The highest confidence structure out of 5 was chosen for each of the peptides.

## Electronic supplementary material

Below is the link to the electronic supplementary material.

Supplementary Material 1

## Data Availability

The data that support the findings of this study are available from the corresponding author upon reasonable request.
