# Supplementary material for: Engineered phages for selective adsorption of rare earth elements
Source: Sci Rep. 2025 Jul 2;15:23549. doi: 10.1038/s41598-025-07604-3 (PMC12223077; doi:10.1038/s41598-025-07604-3)

**Title:** Engineered phages for selective adsorption of rare earth elements

**Authors:** Alexander M. Ditzel^1^, Scott N. Dean^2^, Ellen R. Goldman^2^, and Jinny L. Liu^2*^

^1^ Postdoctoral Fellowship for American Society for Engineering Education.

^2^US Naval Research Laboratory, Center for Biomolecular Science and Engineering, 4555 Overlook Ave., Washington, DC 20375.

*Corresponding author: Jinny.l.liu.civ@us.navy.mil.

**Supplementary information**


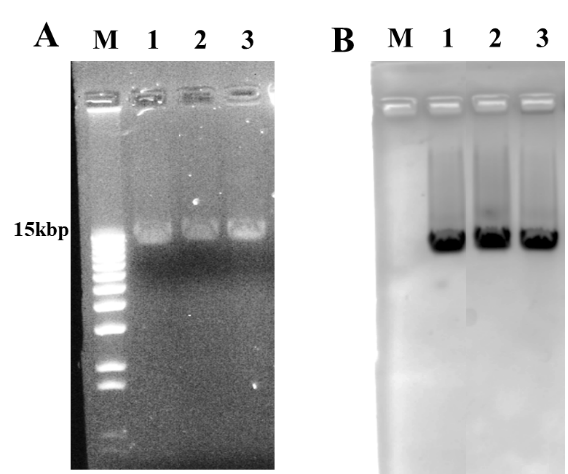


Fig. S1. Purified recombinant phages on native agarose gel. Lanes 1-3 represent H11G-p8, E1-p8 and p8 phages in A and B . A. 1% TAE agarose gel stained with cyber safe DNA dye. B. The same gel stained with Coomassie blue.


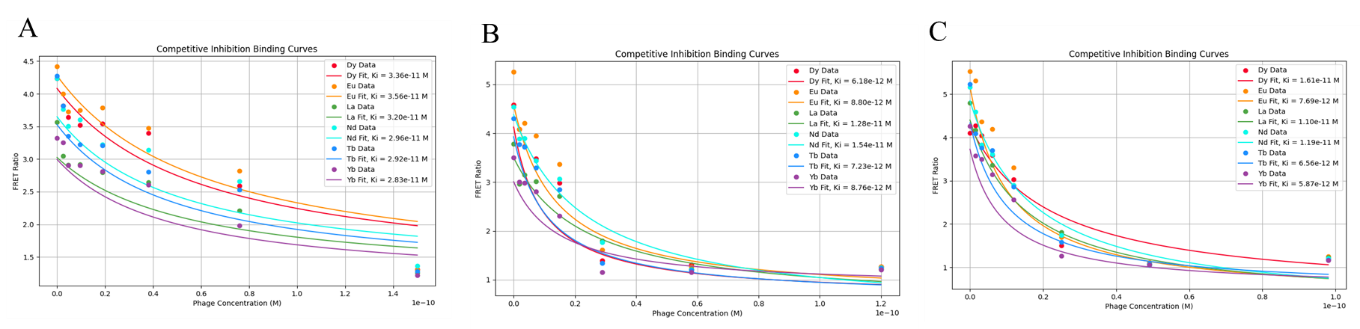


Fig. S2. Ki measurements for a panel of 6 REEs using fitted model with 4 parameters. A. p8 phages. B. H11G-p8 phages. C. E1-p8 phages. Each point was obtained from duplicate or triplicate.


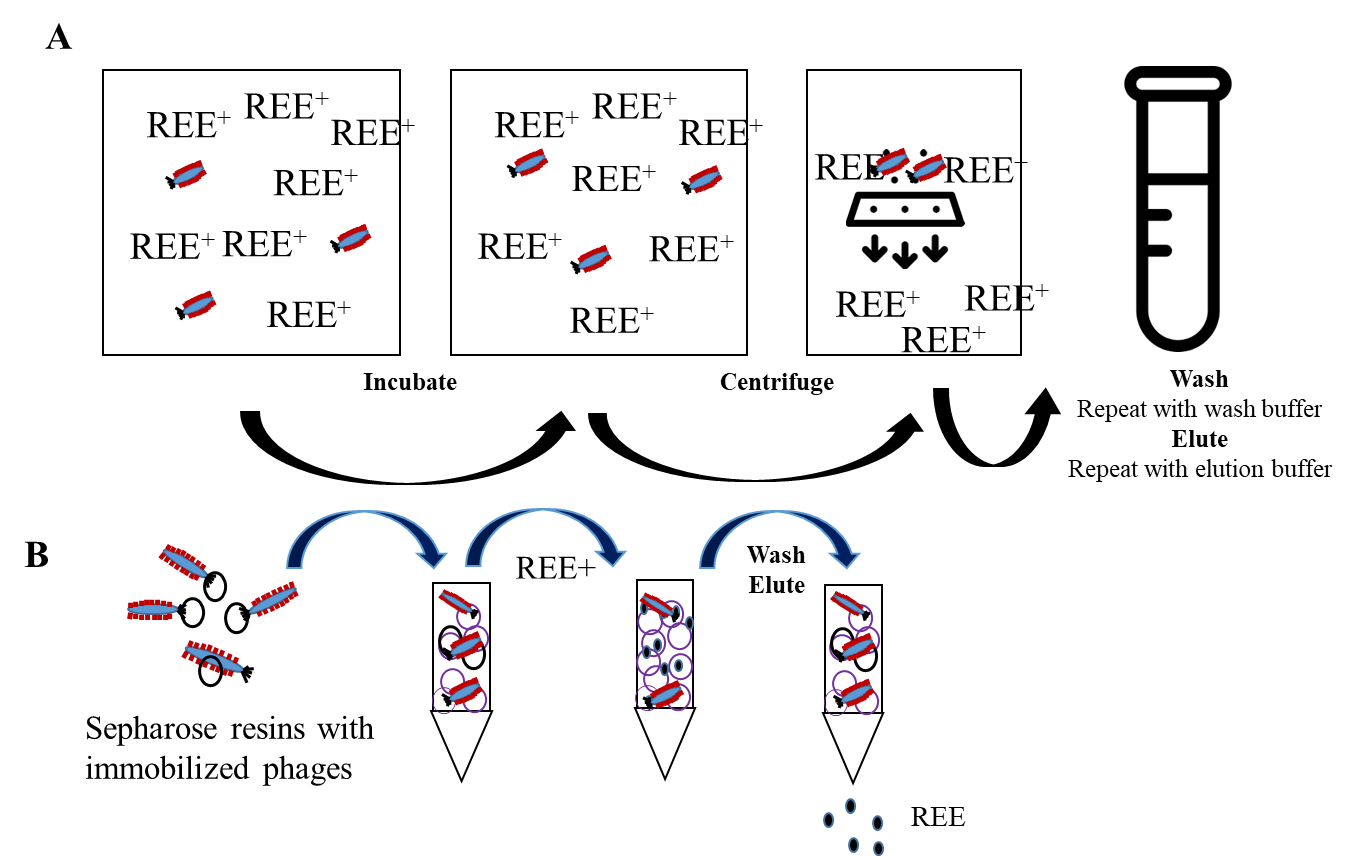


Fig.S3. Method schemes. A. Phage direct binding filter assay. B. REE binding and elution from immobilized phages on sepharose resins.


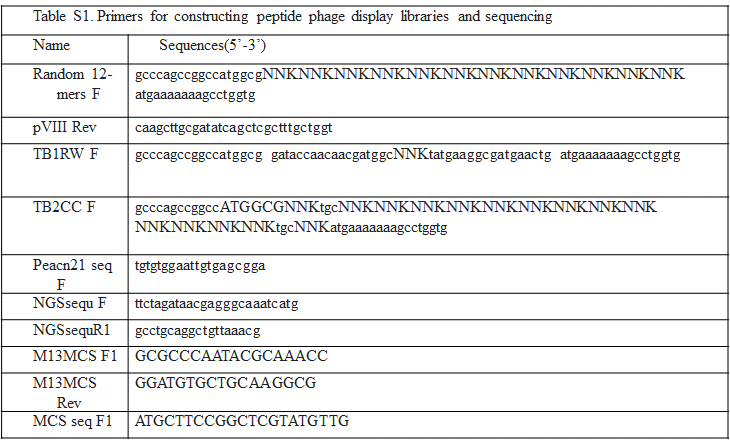

Supplement: Supplementary file 1 — Supplementary Material 1 [file 41598_2025_7604_MOESM1_ESM.docx]
